# Lek Habitat Selection by Sympatric Manakin Species in Northwestern Ecuador

**DOI:** 10.1002/ece3.70860

**Published:** 2025-03-07

**Authors:** Erin Sheehy, H. Luke Anderson, Luis Carrasco, Jorge Olivo, Domingo Cabrera, Nelson Gonzalez, Renata Ribeiro, Jordan Karubian

**Affiliations:** ^1^ Department of Ecology and Evolutionary Biology Tulane University New Orleans Louisiana USA; ^2^ Fundación Para la Conservación de los Andes Tropicales Marcos Jofre Oe5‐227 y Esteban de la Rosa, Edificio Caralis Quito Pichincha Ecuador

**Keywords:** habitat selection, hotspot hypothesis, leks, light environment, niche partitioning, Pipridae

## Abstract

Habitat selection plays a fundamental role in determining community structure and species coexistence, although the role played by sexual selection in shaping settlement patterns is less well understood. Manakins (Pipridae) are a Neotropical family of lekking birds that exhibit similar behavioral ecology across species, both in terms of resource use and dependence on elaborate visual signaling for mate attraction, yet they differ in the form of their sexually selected displays and ornaments. We characterized and compared the spatial dispersion and habitat attributes of lek sites for four species of sympatric manakins in the Chocó region of northwestern Ecuador to test several hypotheses for habitat selection and lek dispersion. First, the interspecific hotspot hypothesis predicts that if males establish leks in locations where females are likely to be encountered (e.g., resource‐rich patches, topographic channels), then leks of ecologically similar species should cluster in geographic space due to shared patterns of resource use among species. Alternatively, the habitat partitioning hypothesis predicts leks of ecologically similar species to exhibit uniform spatial distributions to minimize competition for shared resources. Finally, the signal enhancement hypothesis proposes that males should establish leks in habitats with ambient light or structural properties optimal for the transmission or production of species‐specific mating signals, and thus leks of different species should segregate in environmental space. We found that leks of sympatric manakin species were randomly distributed in geographic space, inconsistent with the interspecific hotspot and habitat partitioning hypotheses. In addition, manakin species segregated in environmental space based on forest structure characteristics related to visual signaling. These findings suggest that landscape‐level lek site dispersion by sympatric manakins may be primarily influenced by sexual display optimization rather than mechanisms related to their shared ecology. Moreover, this study flags the local population of *Masius chrysopterus* as a potential conservation concern due to its distinct and limited elevational preferences.

## Graphical abstract

We aimed to gain insight into the mechanisms driving habitat partitioning among sympatric species subject to strong sexual selection using a community of manakins in northwestern Ecuador. We tested competing hypotheses for the factors driving lek habitat selection and dispersion—(1) the interspecific hotspot hypothesis, which predicts ecologically similar species to establish leks in geographic proximity due to similar patterns of resource use or female movement among species; (2) the habitat partitioning hypothesis,which predicts species' leks exhibit uniform spatial distributions to avoid conflict over shared resources; and (3) the signal enhancement hypothesis, which predicts species to establish leks in environmentally distinct sites to optimize the production or transmission of species‐specific sexual signals— and found stronger evidence for the third hypothesis.

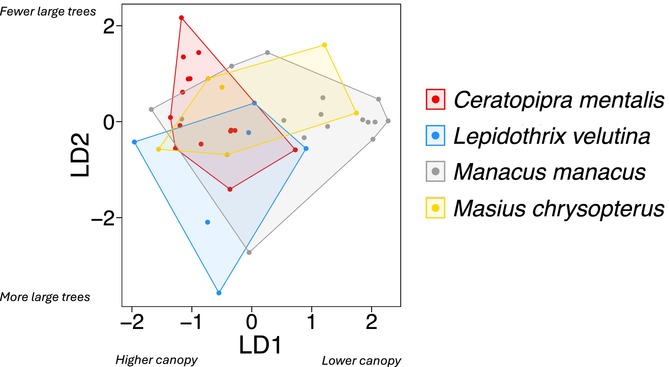

## 1 Introduction

Habitat selection is an important ecological and evolutionary phenomenon that influences population dynamics and community structure (Morris 2003). Organisms are generally thought to select habitats that maximize their fitness (McLoughlin et al. 2006), and when species overlap in physical or niche space, competitive interactions or habitat partitioning may occur (Morris 2003). Habitat selection can also have direct and indirect consequences for mating success that influence the evolutionary trajectories of mating systems (Prum 1990; Hingrat et al. 2007). As such, recognizing the factors that influence habitat selection is crucial to understanding species' behavioral ecology and coexistence.

Lek‐mating species—wherein males aggregate in fixed locations (‘leks’) to perform elaborate courtship displays, and females visit these sites to assess potential mates (Höglund and Alatalo 1995)—provide a useful venue to better understand the interface between sexual selection, niche partitioning, and habitat selection. The stability of leks at specific locations through time (Durães, Loiselle, and Blake 2008; Schlinger, Day, and Fusani 2008) renders the habitat characteristics at these sites particularly important for both near‐term male mating success and long‐term trajectories of signal evolution (Foster 1983; Prum 1990). Numerous hypotheses have been proposed to explain the evolutionary origin and maintenance of leks (e.g., Bradbury 1981; Bradbury and Gibson 1983; Beehler and Foster 1988), and one of these hypotheses—the “hotspot” hypothesis (Bradbury and Gibson 1983)—also has important implications for the placement of leks in space. The hotspot hypothesis proposes that males form display aggregations in areas of high female traffic (e.g., near resource‐rich patches or along environmental features that channel female movement) to increase their probability of encountering females (Bradbury and Gibson 1983). In addition, males may also establish display sites near food resources to increase their own foraging efficiency or energy budgets for display (Anderson, Cabo, and Karubian 2024; Tori 2008). The “interspecific hotspot” hypothesis (Westcott 1994) extends this logic to a multi‐species framework, supposing that if females of ecologically similar, sympatric species exploit the same resources and travel similar routes through the environment, then males of these species should form leks in similar locations (Westcott 1994; Loiselle, Blake, et al. 2007). Thus, the interspecific hotspot hypothesis predicts leks of different species to broadly cluster in geographic space (i.e., the “leks of leks” phenomenon observed in Westcott 1994), and such spatial proximity would result in overlap in environmental space (Loiselle, Blake, et al. 2007). Alternatively, if competition for common resources drives dispersion and partitioning of space among species, leks may be expected to exhibit a uniform distribution across the landscape (i.e., the “habitat partitioning” hypothesis).

In contrast to these resource‐based mechanisms for lek site selection and dispersion, a large body of work on signal evolution suggests that males should select display sites in habitats that optimize the transmission and perception of their mating signals (Endler and Théry 1996; Heindl and Winkler 2003a; Menezes and Santos 2020). This “signal enhancement” hypothesis predicts males of sympatric species with spectrally distinct plumage ornaments to select environmentally distinct habitats for display, as structurally and vegetatively different habitats should generate light environments that differentially impact the transmission and perception of visual signals (Endler 1993; Endler and Théry 1996; Uy and Endler 2004; Uy and Stein 2007). Moreover, independent of light environment, species may also require distinct substrates to perform displays (e.g., fallen logs, vertical saplings, horizontal branches), which may occur more frequently in particular habitat types. Thus, the interspecific hotspot, habitat partitioning, and signal enhancement hypotheses make divergent predictions about the broad spatial and environmental patterns of lek placement among sympatric, ecologically similar species (Table 1). While these mechanisms are not mutually exclusive—for example, resource availability and light environment may both play a role in the placement of lek sites—assessing patterns of lek placement by sympatric lekking species can provide insight into the relative importance of these factors for habitat selection and niche partitioning (Loiselle, Blendinger, et al. 2007), as well as the ways in which sexual selection may shape these ecological processes.

**TABLE 1:** Hypotheses for the factors driving manakin lek placement among sympatric species, their associated predictions for lek dispersion in environmental and geographic space, and the results of this study.

Hypothesis	Mechanism	Space	Prediction	Supported?
Interspecific hotspot	Ecologically similar species place leks on shared resource hotspots or locations where female movements are similarly channeled	Geographic	Clustered	No
Habitat partitioning	Ecologically similar species maximize distances between lek sites to reduce competition for shared resources		Uniform	No
Null (non‐resource‐related mechanisms)	If the above resource‐related mechanisms are relatively unimportant, dispersion of heterospecific leks may not differ from complete spatial randomness		Random	Yes
Interspecific hotspot	Geographic clustering around female hotspots also results in overlap in environmental space	Environmental	Overlap	No
Signal enhancement	Males establish leks in locations that optimize transmission or production of species‐specific visual signals		Separation	Yes

Manakins (Pipridae) are a family of Neotropical lek‐mating birds known for their elaborate male plumage and mating displays (Prum 1994). They are abundant in the American tropics, with 16 named species of manakins in Ecuador alone, where many species co‐occur locally (Loiselle, Blendinger, et al. 2007). Notably, a previous study by Loiselle, Blake, et al. (2007) demonstrated that six sympatric manakin species in the Ecuadorian Amazon form leks in geographically and environmentally distinct microhabitats, generally segregating by elevation and slope. However, this study focused on ‘macroscale’ topographic variables and did not consider habitat variables expected to correlate with light environment per se, such as canopy height and openness. Another study examining four species of manakins in the Venezuelan Amazon demonstrated that males display in forest strata that maximize the reflectance and contrast of species‐specific plumage elements (Heindl and Winkler 2003b). To date, this work on Amazonian manakins is inconsistent with sympatric species converging on similar lek habitats as predicted by the interspecific hotspot hypothesis, instead suggesting that different species select distinct habitat characteristics for display, as predicted by the signal enhancement hypothesis. Additional studies of sympatric manakin species in other biogeographic zones can provide insight into the generality of these patterns.

**FIGURE 1:**
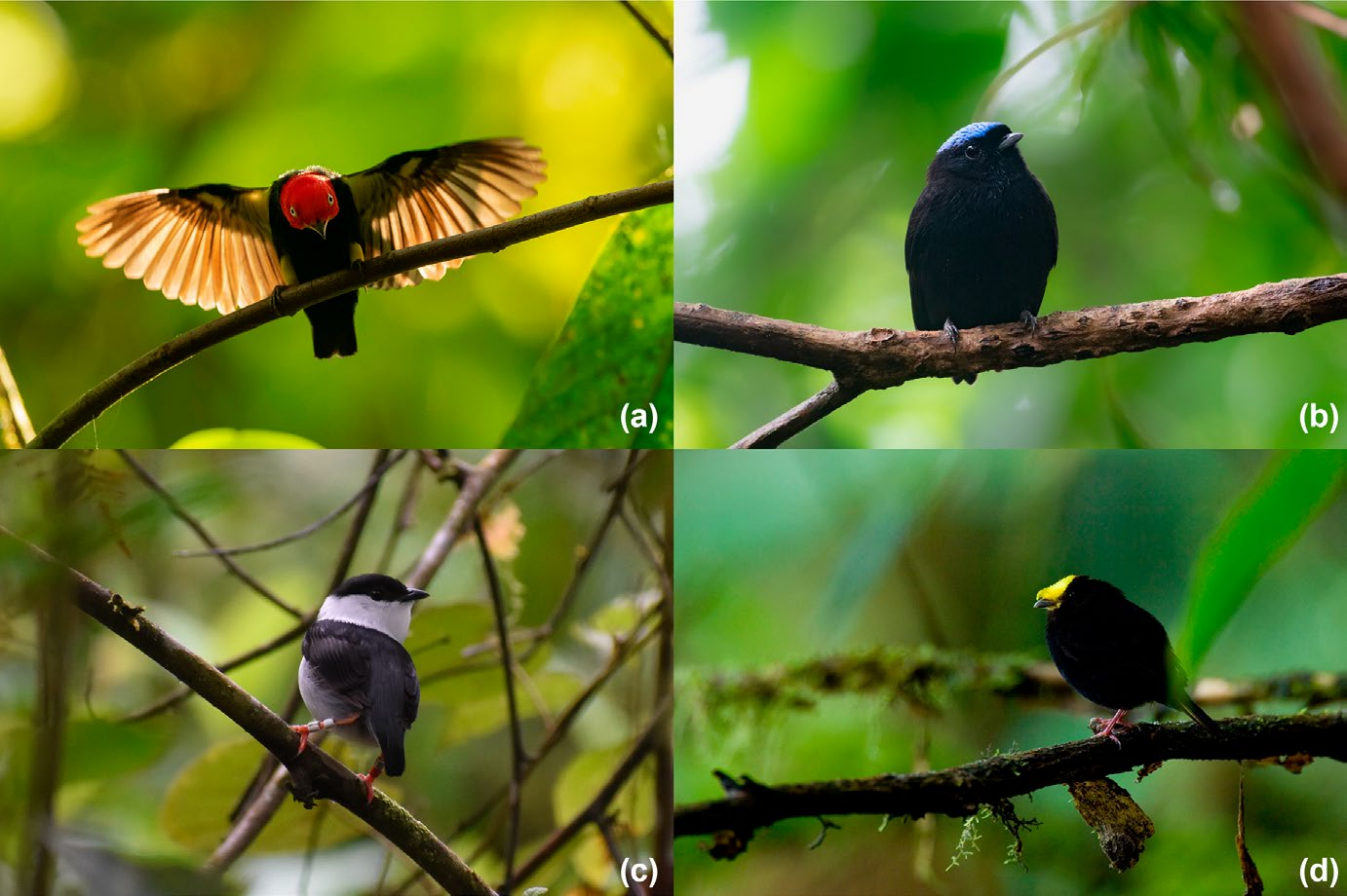
Males of the four focal manakin species represented in this study. (a) Red‐capped manakin, *Ceratopipra mentalis* (Photo: Murray Cooper). (b) Velvety Manakin, *Lepidothrix velutina* (Photo: Brandon Nidiffer; Cornell Lab of Ornithology|Macaulay Library). (c) White‐bearded Manakin, *Manacus manacus* (Photo: Samuel B. Case). (d) Golden‐winged Manakin, *Masius chrysopterus* (Photo: Murray Cooper).

We examined the environmental correlates of lek site selection in four species of sympatric manakins in the Chocó rainforests of northwestern Ecuador: the red‐capped manakin (*Ceratopipra mentalis*), velvety manakin (*Lepidothrix velutina*), white‐bearded manakin (*Manacus manacus*), and golden‐winged manakin (*Masius chrysopterus*; Figure 1). Notably, all four species are characterized by elaborate male courtship displays and marked sexual dichromatism, featuring distinct color patches in their definitive male plumage. The four species also use distinct substrates to perform the most iconic elements of their display repertoires: *M. manacus* males leap between vertical saplings (Snow 1962); *L. velutina* males perform butterfly‐like flights between horizontal perches in the understory (Durães 2009); *C. mentalis* males slide along horizontal branches in the subcanopy (Skutch 1949); and *M. chrysopterus* males flip acrobatically on fallen logs and exposed buttress roots (Prum and Johnson 1987). One species (*M. manacus*) forms concentrated leks, with males potentially displaying in both acoustic and visual contact of one another (Snow 1962), while the others (*C. mentalis*, *L. velutina*, and *M. chrysopterus*) typically form dispersed leks, with males displaying in auditory but not visual contact (Skutch 1949; Prum and Johnson 1987; Prum 1994; Durães 2009); however, we note that species' leks can vary in their degree of dispersion within and between populations. Importantly, all four species feed generally on the same fruit species (J. Karubian and J. Olivo, unpublished data), making this a useful system for investigating patterns of lek site selection and habitat partitioning among ecologically similar species that differ in sexual display characteristics (Loiselle and Blake 1999).

We reasoned that if these ecologically similar species select lek sites based primarily on resource availability or environmental channeling as predicted by the interspecific hotspot hypothesis, lek sites of the four species should be clustered in geographic and environmental space due to their shared patterns of resource use. Alternatively, if habitat partitioning occurs to avoid competition for common resources, lek sites might be expected to exhibit a uniform distribution in geographic space. If resource‐related mechanisms are not influential in shaping the dispersion of interspecific lek sites, then leks of sympatric species would not be expected to exhibit strong clustering or uniformity in geographic space, and heterospecific lek distributions would be indistinguishable from complete spatial randomness. Finally, although we did not measure light environment directly, we hypothesized that if manakins select lek sites based primarily on habitat characteristics related to species‐specific visual signaling (i.e., components of three‐dimensional forest structure), the four species' lek sites should largely segregate in environmental space due to their spectrally distinct male plumage patches. In addition to testing these hypotheses for lek placement (Table 1), we aimed to determine the degree to which patterns of lek habitat segregation among manakins in the Amazon (observed by Loiselle, Blake, et al. 2007) are borne out in manakin communities in an adjacent but biogeographically distinct region, the Chocó. Importantly, assessing species' habitat‐level display site requirements may also be useful in identifying species or populations of conservation concern, as the breadth and vulnerability of the preferred lek habitats for a given species may indicate its resilience to continued anthropogenic habitat modification and climate change in tropical environments.

## 2 Methods

### 2.1 Study Area

We conducted this study in the Reserva Ecológica Mache Chindul (REMACH), Esmeraldas Province, Ecuador, located in the Chocó biogeographic zone. The Chocó is a top biodiversity hotspot globally, with high levels of diversity, endemism, and habitat loss (Myers et al. 2000). Despite being of high conservation priority, this ecosystem is greatly understudied. Research was focused at two sites: the Fundación para la Conservación de los Andes Tropicales Reserve (FCAT, 0°22.387′ N, 79°39.919′ W; 550 ha) and Bilsa Biological Station (BBS, 0°20.816′ N, 79°42.659′ W; 3500 ha). These sites are located in and around the Mache Chindul Ecological Reserve, which has suffered high rates of ongoing deforestation in the past 50 years and contains a mixture of intact pre‐montane forest, early successional forest, and pastureland (Van Der Hoek 2017; Kleemann et al. 2022). In July and September of 2020, we exhaustively searched the entire FCAT Reserve and approximately the northeast quadrant of BBS for manakin leks. During these surveys, we noted the location of new leks, confirmed activity of previously identified leks, and gathered relevant environmental data (below).

### 2.2 Data Collection

We identified male display areas within a lek by selecting a specific location (i.e., tree branch or display court) where an adult male was observed actively displaying on at least two separate occasions separated by at least 1 week during the three‐month sampling period. Entire leks were not mapped, and thus we do not have information about the total lek size or the centrality of focal territories. We assumed display courts located > 200 m apart belonged to distinct leks, given that this is approximately twice the typical nearest neighbor distance reported within exploded leks of *Masius* and *Lepidothrix* (Durães 2009; Kirwan and Green 2011) and courts spaced this far apart are likely out of earshot of one another. To avoid pseudoreplication by sampling multiple territories in the same lek, only conspecific territories separated by at least this distance were included in analyses. We acknowledge that data from a single territory may not always be representative of all territories within a given lek, although surveys spanning multiple lek sites should provide a reasonable assessment of overall habitat characteristics for a given species.

We quantified the environmental characteristics at each male display territory by measuring six habitat variables shown in previous studies to be important predictors of species composition and behavior in our project areas (e.g., Durães et al. 2013; Mahoney et al. 2018; Lamperty, Karubian, and Dunham 2021): elevation, canopy openness, canopy height, number of trees with diameter at breast height between 10 and 50 cm (DBH > 10) within a 10 m radius, number of trees with diameter at breast height greater than 50 cm (DBH > 50) within a 20 m radius, and the abundance of the pioneer tree species *Cecropia* within a 10 m radius. We assessed canopy heights using a laser distance measurer. To measure canopy openness, we used a spherical densiometer to quantify the amount of light entering the canopy, with measurements taken in the four cardinal directions and averaged; higher densiometer values indicate greater canopy openness. We note that we did not directly measure light environment in this study and recognize that direct measurement of ambient light spectra at forest strata relevant to a given species' display is required to definitively test hypotheses of lek placement related to visual signaling. However, the variables measured here (i.e., canopy height, canopy openness, DBH > 10, and DBH > 50) enable characterization of three‐dimensional forest structure, which can serve as a useful proxy for ambient light environment and inform predictions of the signal enhancement hypothesis.

We also recorded the number of Melastomataceae and Rubiaceae plants within a 10 m radius of our survey points, as plants in these families produce fruits that comprise large portions of manakin diets (Loiselle and Blake 1990; Loiselle, Blendinger, et al. 2007). However, these data were excluded from analysis because they could not provide clear tests of the hotspot hypothesis; such a test would require comparing on‐lek fruit or fruit plant abundance to off‐lek control sites (per Ryder, Blake, and Loiselle 2006). In addition, the interspecific hotspot hypothesis as posed by Westcott (1994) predicts species to converge on the same resource or movement hotspots, which is better assessed by evaluating the dispersion of lek sites in geographic space.

### 2.3 Statistical Analyses

All analyses were conducted in version 1.2.1335 of R (R Core Team 2023). Per Westcott (1994) and Loiselle, Blake, et al. (2007), we tested for geographic clustering of lek sites within the FCAT Reserve using a Clark‐Evans test with edge correction, which was implemented using the *spatstat.explore* package in R (Baddeley 2024). The test uses nearest‐neighbor distances to assess whether points exhibit clustered or uniform dispersion relative to a null hypothesis of complete spatial randomness (Clark and Evans 1954). Because the Clark–Evans index is relatively crude, we also conducted a maximum absolute deviation (MAD) test to assess whether point dispersion differed from complete spatial randomness across a range of neighborhood sizes, which involves calculating Ripley's *K‐*function for the observed data and comparing it to the simulated confidence envelope for the theoretical null model (*n* = 1,000 simulations). The GPS points of two display sites were located just outside the bounds of the FCAT Reserve, and thus these sites (*n* = 1 *M. manacus*, *n* = 1 *L. velutina*) were excluded from the FCAT spatial analysis. We were not able to perform statistical tests of spatial dispersion for leks within Bilsa Biological Station, as tests require exact survey boundaries to be defined in order to calculate the degree of spatial randomness for point patterns within those bounds, and the precise geographical extent of surveys within BBS was not known.

We next used linear discriminant analysis (LDA) to investigate whether leks of different manakin species overlapped or segregated in environmental space (following Loiselle, Blake, et al. 2007), with a focus on those variables likely to be associated with differences in visual signal transmission. LDA is a form of dimensionality reduction that optimizes the distance between means to maximize the separability of a priori groups (Tharwat et al. 2017), which in this case were the four manakin species. The values for habitat variables obtained for each lek were centered and scaled prior to use in analyses. Of the habitat variables collected at lek sites described above, only those expected to be relevant to the visual signaling hypothesis were used in the LDA. This included canopy height, canopy openness, DBH > 10, and DBH > 50, all of which characterize three‐dimensional forest structure that could influence ambient light environment, available forest strata, and available display substrates. We used Shapiro–Wilk, Mardia, Levene, and Box's M tests, respectively, to evaluate assumptions of within‐class univariate normality, within‐class multivariate normality, between‐class homogeneity of variance, and homogeneity of covariance matrices. The latter three assumptions were met, although some within‐class deviations from univariate normality were present. Thus, to assess the significance of environmental segregation in the lek habitat LDA, we supplemented the results of classical MANOVA with nonparametric comparisons for multivariate data obtained using the R package *npmv* (Burchett, et al. 2017). Parametric and nonparametric approaches to inference yielded similar estimates of significance, and we present both results below.

## 3 Results

**FIGURE 2:**
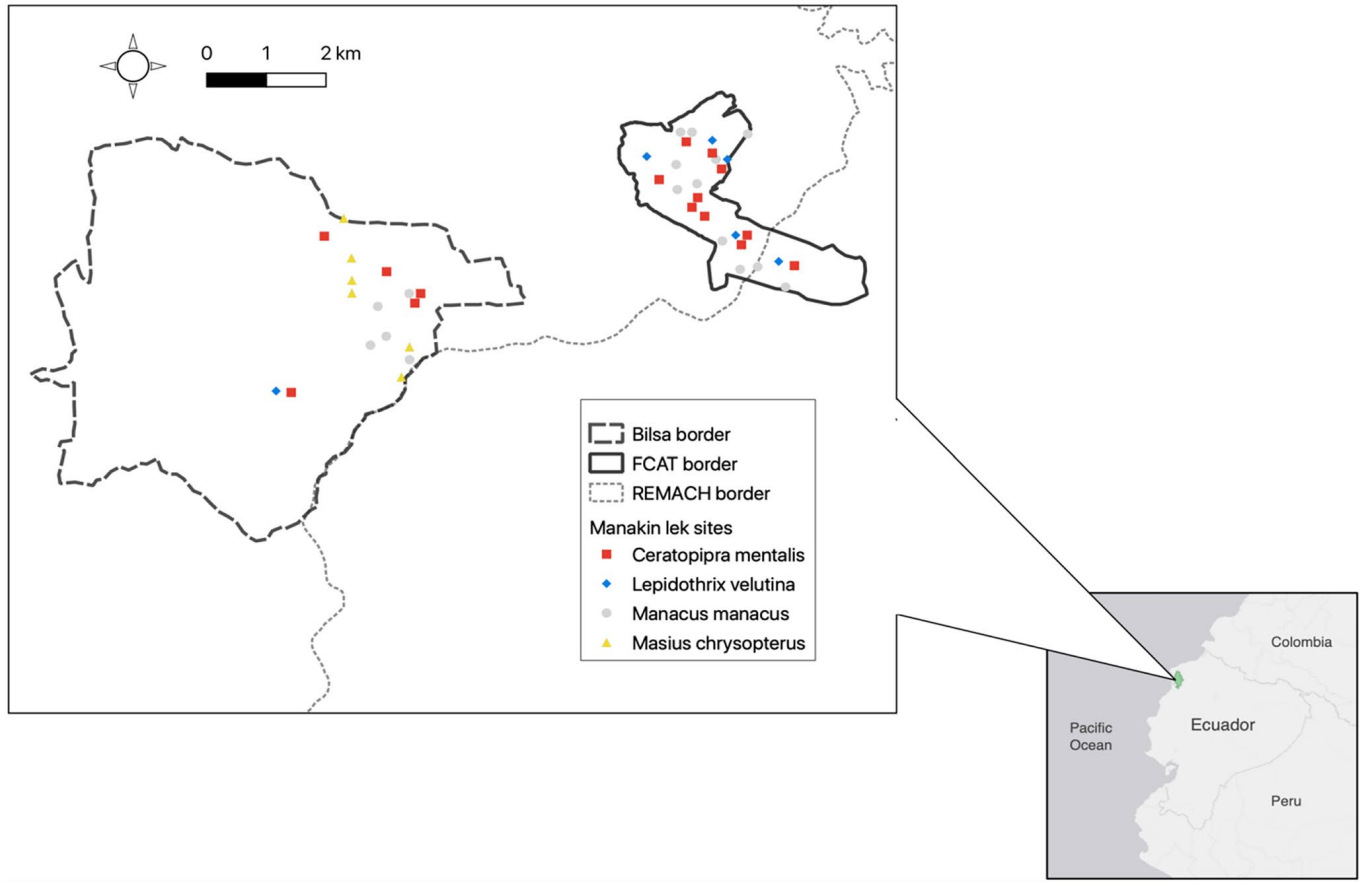
Location of manakin leks on two study plots, Bilsa Biological Station and the FCAT Reserve, within the bounds of the Reserva Ecológica Mache Chindul (REMACH). Inset: Location of REMACH (green shaded area) in the context of Ecuador and northwestern South America.

We collected habitat measures at a total of 43 manakin display sites, each representing a distinct lek. Sites were sampled from a total of 16 *M. manacus* leks, 15 *C. mentalis* leks, six *L. velutina* leks, and six *M. chrysopterus* leks (Figure 2). Broadly, we observed leks of the four species in the following major habitat types, which were determined based on known land‐use history: *C. mentalis* leks occurred in primary forest (86.67%), secondary forest (6.67%), and altered (selectively logged) forest (6.67%); *M. manacus* leks occurred in secondary forest (62.5%) and primary forest (37.5%); *L. velutina* leks occurred exclusively in primary forest (100%); and *M. chrysopterus* leks occurred in primary forest (66.7%) and secondary forest (33.3%).

**FIGURE A1:**
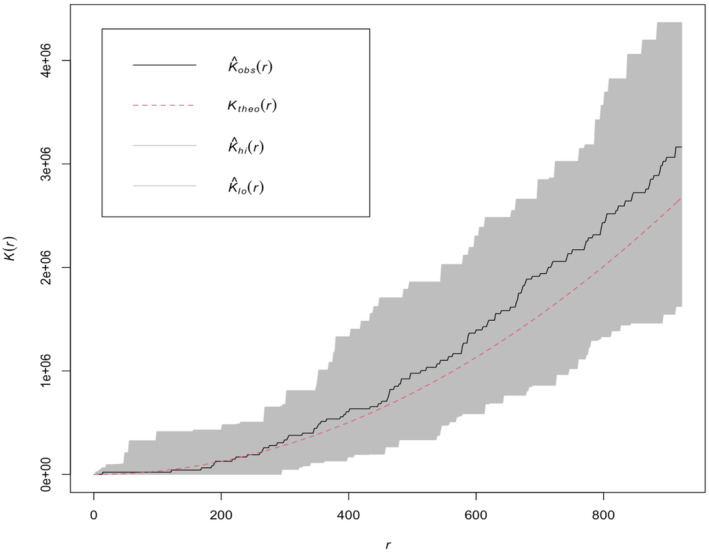
Visualization of Ripley's *K*‐function across multiple scales for lek sites within the FCAT Reserve. The *x* axis represents distance (neighborhood size), and the y axis represents the value of *K* at a given distance (*r*). The red line represents the theoretically expected degree of clustering under complete spatial randomness, the black line represents the observed degree of spatial clustering in the empirical data, and the shaded area represents the 95% confidence envelope for *n* = 1000 simulations of *K*(*r*) under complete spatial randomness.

Within the FCAT Reserve, the spatial distribution of lek locations across species did not significantly differ from complete spatial randomness based on a Clark–Evans test (*R* = 0.99, *p* = 0.95). We note that *M. chrysopterus* did not occur within FCAT, and thus this result is limited to only the other three species. In addition, the observed dispersion of lek sites across the three species at FCAT did not differ from complete spatial randomness at any spatial scale based on the Ripley's *K‐*function (MAD = 541,203; rank = 196, *p* = 0.20; Appendix Figure A1), providing no evidence for spatial clustering or uniformity of heterospecific lek sites as would be predicted by the interspecific hotspot or habitat partitioning hypotheses, respectively.

**FIGURE 3:**
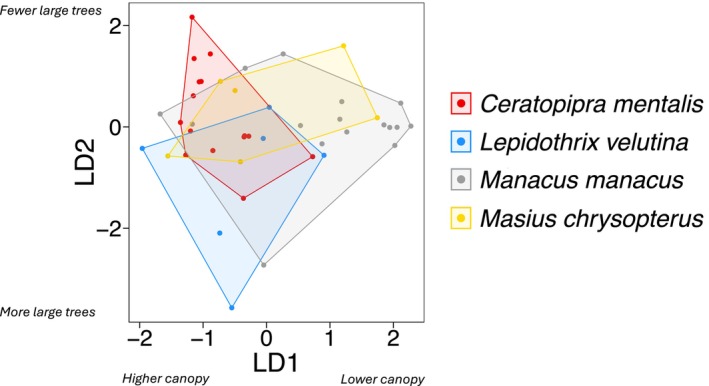
Location of leks (*n* = 43) in environmental space for four sympatric manakin species in northwest Ecuador, based on discriminant function analysis using habitat variables assumed to be related to visual signaling. Variables that loaded most strongly along each linear discriminant (canopy height and number of trees with DBH > 50 cm, respectively) are noted in axis labels. The first linear discriminant function (LD1) explained 66.8% of the between‐class variance, while the second linear discriminant function (LD2) explained 25.5%. Colors correspond to the four manakin species.

The four species exhibited significant separation in environmental space based on putative visual signaling variables (MANOVA: Pillai's trace = 0.62, df = 3, *p* < 0.01; Wilks' lambda = 0.48, df = 3, *p* < 0.01; nonparametric multivariate inference, F‐approximation for Wilks' lambda: F_12, 95.5_ = 2.33, *p* = 0.01). Linear discriminant analysis identified canopy height and number of large trees (i.e., DBH > 50 cm) as particularly salient variables differentiating the lek habitat characteristics of our study species (Table 2; Figure 3; Appendix Table A1). The first linear discriminant function (LD1) explained 66.8% of the between‐group variation and primarily separated leks by canopy height, while the second linear discriminant (LD2) explained 25.5% of the between‐group variation and primarily separated leks primarily based on number of large trees (i.e., DBH > 50 cm).

**TABLE 2:** Linear discriminant function loadings. Proportion of between‐class variance explained by each linear discriminant function is indicated by the proportion of trace.

	LD1	LD2	LD3
Canopy height (m)	−1.12	0.76	0.32
# large trees (DBH > 50 cm)	−0.35	−1.13	0.01
# small trees (DBH 10–50 cm)	−0.29	0.57	−0.48
Canopy openness	−0.28	0.12	0.85
Proportion of trace	0.67	0.25	0.08

**FIGURE 4:**
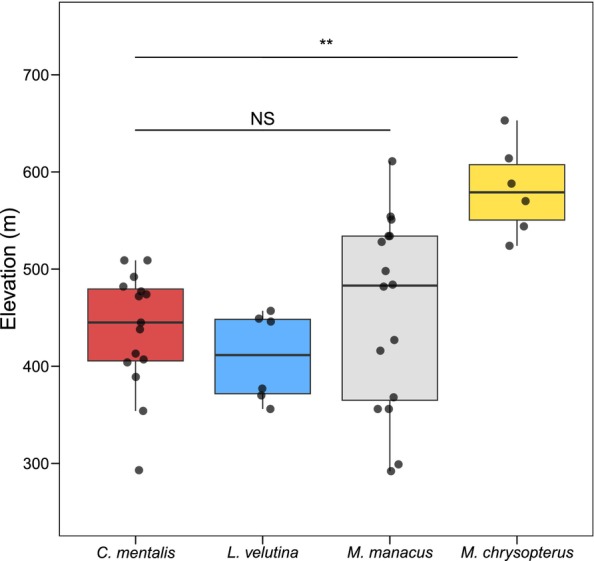
Elevational differences in lek habitat selection among manakin species. Based on an ANOVA with pairwise Tukey's post hoc tests, lek sites of *M. chrysopterus* occurred at significantly higher elevations than leks of the other three species (** = adjusted *p* < 0.01). No significant pairwise differences in lek site elevation were observed among the other three species.

Using the jackknife (i.e., “leave‐one‐out”) prediction method, the LDA correctly classified 60.5% of the 43 leks to species based on environmental correlates. Leks of *C. mentalis* were classified with 86.7% accuracy, *M. manacus* with 68.8% accuracy, *L. velutina* with 33.33% accuracy, and *M. chrysopterus* with 0.0% accuracy. The lower classification accuracy for *L. velutina* and *M. chrysopterus* lek sites may be related to the smaller sample sizes for these species. *M. manacus* occupied a relatively large environmental space, although display sites tended to occur in areas with lower canopy heights and fewer large trees. Leks of *C. mentalis* and *L. velutina* tended to occur in locations with taller canopies and, in the case of *L. velutina* , greater numbers of large trees. *M. chrysopterus* leks exhibited considerable variance in signal‐related variables and were not accurately classified by the jackknife procedure. Importantly, however, lek sites of this species occurred at significantly higher elevations than leks of all other species (Figure 4; Table 3).

**TABLE 3:** Results of pairwise Tukey's post hoc tests comparing lek site elevations among the four manakin species.

	*L. velutina*	*M. manacus*	*M. chrysopterus*
*C. mentalis*	*p* = 0.87	*p* = 0.90	*p* = 0.001**
*L. velutina*	—	*p* = 0.57	*p* = 0.002**
*M. manacus*	—	—	*p* = 0.006**

*Note:* Values of *p* are adjusted for multiple comparisons.

** *p* < 0.01.

## 4 Discussion

Resolving the ways in which lek sites of closely related species segregate in relation to environmental variables allows for better understanding of the factors shaping the evolutionary ecology of lek mating systems and, more broadly, the coexistence of ecologically similar species. Here, we characterized lek spatial dispersion and habitat attributes for sympatric manakin species with similar foraging ecologies in northwest Ecuador. In contrast to both the interspecific clumping (i.e., “leks of leks” phenomenon) observed by Westcott (1994) and the uniform spatial dispersion of leks of six sympatric manakin species observed by Loiselle, Blake, et al. (2007), the geographic distribution of manakin lek sites of the three species present within the FCAT Reserve did not differ significantly from complete spatial randomness. However, we did observe significant between‐species segregation of lek sites in environmental space based on variables potentially related to visual signaling, including canopy height and number of large trees (i.e., DBH > 50 cm). While this study primarily investigated structural characteristics of forest habitats (e.g., canopy height, openness, tree size) and Loiselle, Blake, et al. (2007) assessed more ‘macroscale’ topographic features (e.g., slope, elevation, distances to rivers), both studies obtained qualitatively similar results: sympatric manakins exhibit considerable interspecific segregation in lek habitat selection that is inconsistent with the predictions of the interspecific hotspot hypothesis (Westcott 1994). Instead, results are consistent with the hypothesis that species‐specific habitat preferences or requirements govern lek placement and influence landscape‐level habitat selection and partitioning in sympatric manakins.

Although we did not take direct measurements of ambient light environment in this study, segregation of species in environmental space is consistent with the possibility that light environment is an important determinant of lek placement due to its influence on plumage reflectance spectra, contrast of sexually selected ornaments from the background environment, and subsequent visual signal transmission (Endler and Théry 1996; Heindl and Winkler 2003b; Uy and Stein 2007). Canopy height, which influences the available display strata and thus the ambient light spectra (Endler 1993), was the variable that most strongly separated species' lek sites in environmental space. Known differences in the height of display perches across genera—with *Ceratopipra* preferring to display in the midstory and subcanopy (typically 5–15 m; Kirwan and Green 2011), *Lepidothrix* in the understory (typically 3–4 m; Kirwan and Green 2011), and *Manacus* and *Masius* very close to the ground (on saplings or fallen logs and buttress roots, respectively; Kirwan and Green 2011)—are consistent with the idea that each species may display in forest strata characterized that enhance species‐specific color patches. For example, higher forest strata have been shown to enhance the chromatic contrast of red and yellow plumage patches characteristic of *C. mentalis* , while lower forest strata can enhance the chromatic contrast of blue plumage patches characteristic of *L. velutina* (Heindl and Winkler 2003b).

However, there are also reasons to be skeptical of the signal enhancement hypothesis. Across all three linear discriminant axes, species did not strongly segregate by canopy openness (Table 2), which was the variable expected a priori to correlate most strongly with ambient light environment. One possible explanation for this pattern is that the four species may experience similar (perhaps predator‐related) constraints on lek placement, leading all to prefer relatively closed canopies. Nonetheless, canopy height and other structural attributes of habitat did vary across species, and there are many other fine‐scale features of forest light environments that we did not explore in this study, such as reflectance spectra measurements, vegetation geometry, sun angle, and weather (Théry 2001). Building on previous studies that have examined the spectral composition of various manakin plumages in their display environments (e.g., Endler and Théry 1996; Heindl and Winkler 2003a, 2003b; Uy and Endler 2004; Doucet, Mennill, and Hill 2007; Uy and Stein 2007), obtaining direct spectral measurements of each manakins' plumage in their respective display microhabitats is a key next step toward understanding the importance of light environment in shaping interspecific habitat selection, lek dispersion, and niche partitioning in our system, particularly given that light environments can vary even within habitat types due to fine‐scale differences in forest geometry (Endler 1993).

In addition to light environment, other unmeasured environmental factors associated with visual displays may have also varied between species' lek sites. The requisite display substrates for a given species are likely to be more abundant in certain habitat types than others. For instance, *M. manacus* males may be more likely to encounter vertical saplings in regenerating secondary forest (characterized by lower canopy height), while *C. mentalis* males may more frequently encounter high horizontal branches in older‐growth forest (characterized by higher canopy height). Measuring habitat variables directly associated with display production, as well as understanding the degree to which males modify their display performance or repertoire when ideal habitat or display substrates are limited, represents another opportunity for future research. In addition, it would be interesting to explore how the tendency for lek sites to remain traditional in location over many years intersects with the dynamic nature of anthropogenic habitat modification and forest succession.

While the broad‐scale patterns of lek placement we observed are inconsistent with the idea that ecologically similar lekking species converge geographically or environmentally on resource‐rich areas as predicted by the interspecific hotspot hypothesis (Table 1), this does not preclude hotspot mechanisms from operating within species and habitat types. Indeed, when fruit availability and biomass were directly measured in Amazonian manakins, the leks of several species found to segregate environmentally in Loiselle, Blake, et al. (2007) were shown to occur in areas of higher fruit availability relative to non‐lek control areas (Ryder, Blake, and Loiselle 2006); this appears to be the case for *M. manacus* leks at our study site as well (Casement 2022), and *Manacus* display courts surrounded by greater average fruit biomass were found to exhibit higher rates of male display and female visitation at our site (Anderson, Cabo, and Karubian 2024). A possible explanation for these seemingly contradictory findings is that habitat selection occurs via a hierarchical process, wherein the spatial partitioning of display sites among sympatric species may be primarily attributable to display‐related habitat characteristics (e.g., available substrates, ambient light conditions), but males of a given species form leks on resource hotspots within preferred habitat types. Additionally, when ecologically similar species occur in sympatry, they often adopt distinct patterns of resource and microhabitat use (e.g., for nesting, resting, bathing; Bazzaz and Catovsky 2001), which in turn could produce distinct patterns of female traffic between species and result in non‐overlapping female movement hotspots. Therefore, while the segregation of leks in environmental space observed in this study and in Loiselle, Blake, et al. (2007) is inconsistent with shared resource hotspots driving spatial convergence of lek habitat selection among sympatric manakin species, we cannot rule out the possibility that resource or female traffic hotspots influence lek placement within individual species. Alternatively, it may be that foraging niches of these sympatric species differ in ecologically important ways in our study area. As such, directly measuring species‐specific resource distributions (Ryder, Blake, and Loiselle 2006) and female movement patterns (Durães, Loiselle, and Blake 2007) represents another opportunity for future research in this system.

Finally, we note that the low abundance of *Masius chrysopterus* leks at our study site, combined with its apparent elevational restriction (Figure 4), suggests reason for conservation concern for this population. Deforestation occurs at a high rate within the Mache Chindul Reserve (Van Der Hoek 2017; Kleemann et al. 2022), and *M. chrysopterus* lek sites are already confined to some of the highest points in the Mache Chindul Cordillera (maximum 700 m asl, and a large majority < 600 m asl), which is isolated from the Andes proper. If habitat loss in this area continues, this population of *M. chrysopterus* does not have the option of upslope migration. Such a situation, combined with warming temperatures, poses a major extinction risk for elevationally restricted taxa (Sekercioglu et al. 2008; Freeman et al. 2018). Longitudinal mist‐netting data from our site indicate that *M. chrysopterus* is among the species most negatively impacted by fragmentation in the area (with strong negative effects also noted for *C. mentalis* and *L. velutina* ), based on decreases in species abundance in forest fragments compared to adjacent contiguous forest (Ellis 2024). In contrast, *M. manacus* leks occupied the widest swath of environmental space, including sites with relatively low canopy heights and high numbers of Cecropia (Appendix Table A1), suggestive of greater resilience to ongoing habitat modification. This interpretation is also corroborated by mist‐netting data, with *M. manacus* numbers generally increasing with fragmentation at our site (Ellis 2024). The concordance between empirical abundance estimates and lek habitat restriction suggests the potential value of assessing species‐specific habitat preferences in the context of sexual selection per se, in addition to the nesting and foraging requirements typically considered in conservation plans and assessments. Overall, this study demonstrates that considering spatial dispersion and three‐dimensional forest structure can be useful for understanding the mechanisms shaping species' ecology, habitat selection, and coexistence, and suggests avenues for future work to better understand the degree to which ecological versus sexual selection pressures may be shaping population trends for this iconic component of the neotropical avifauna.

## Author Contributions

**Erin Sheehy:** conceptualization (equal), data curation (equal), project administration (equal), writing – original draft (lead), writing – review and editing (equal). **H. Luke Anderson:** conceptualization (equal), data curation (equal), formal analysis (lead), methodology (equal), project administration (equal), software (lead), supervision (supporting), validation (lead), visualization (lead), writing – review and editing (lead). **Luis Carrasco:** conceptualization (equal), investigation (equal), project administration (supporting). **Jorge Olivo:** investigation (equal), methodology (equal). **Domingo Cabrera:** investigation (equal), methodology (equal). **Nelson Gonzalez:** investigation (equal), methodology (equal). **Renata Ribeiro:** conceptualization (equal), methodology (equal), supervision (equal), writing – review and editing (equal). **Jordan Karubian:** conceptualization (equal), methodology (equal), project administration (equal), resources (lead), supervision (equal), writing – review and editing (equal).

## Conflicts of Interest

The authors declare no conflicts of interest.

## Data Availability

All data and code files are publicly available on Dryad, accessible at the following link: https://doi.org/10.5061/dryad.ncjsxkt4c.

## Appendix A

**TABLE A1:** Summary statistics for environmental data collected at manakin display sites.

	*C. mentalis*	*L. velutina*	*M. manacus*	*M. chrysopterus*
	Mean SD	Mean SD	Mean SD	Mean SD
Elevation (m)	**437.20** *61.04*	**409.17** *46.10*	**455.63** *98.30*	**582.17** *47.02*
*Cecropia* (count)	**1.80** *2.11*	**0.50** *1.22*	**2.56** *2.56*	**0.67** *0.82*
# DBH > 10 (cm)	**13.80** *4.47*	**11.33** *3.44*	**12.63** *4.80*	**11.33** *5.92*
# DBH > 50 (cm)	**2.33** *1.11*	**3.33** *1.97*	**1.13** *1.50*	**1.33** *1.37*
Canopy Height (m)	**31.67** *3.46*	**28.83** *4.31*	**23.44** *6.26*	**28.33** *7.74*
Canopy Openness (avg. densiometer)	**4.39** *5.74*	**5.68** *7.49*	**7.87** *9.54*	**10.88** *10.98*

*Note:* Bolded values correspond to means, italicized values correspond to SDs.
